# Impact of ovary-intact menopause in a mouse model of heart failure with preserved ejection fraction

**DOI:** 10.1152/ajpheart.00733.2023

**Published:** 2024-01-05

**Authors:** Aaron M. Troy, Diyora Normukhamedova, Daniela Grothe, Abdul Momen, Yu-Qing Zhou, Meghan McFadden, Mansoor Hussain, Filio Billia, Hai-Ling Margaret Cheng

**Affiliations:** ^1^Institute of Biomedical Engineering, University of Toronto, Toronto, Ontario, Canada; ^2^Translational Biology and Engineering Program, Ted Rogers Centre for Heart Research, Toronto, Ontario, Canada; ^3^Toronto General Hospital Research Institute, University Health Network, Toronto, Ontario, Canada; ^4^Peter Munk Cardiac Centre, University Health Network, Toronto, Ontario, Canada; ^5^Heart and Stroke/Richard Lewar Centre of Excellence in Cardiovascular Research, University of Toronto, Toronto, Ontario, Canada; ^6^Department of Medicine, University of Toronto, Toronto, Ontario, Canada; ^7^Department of Medical Biophysics, University of Toronto, Toronto, Ontario, Canada; ^8^The Edward S. Rogers Sr. Department of Electrical and Computer Engineering, University of Toronto, Toronto, Ontario, Canada

**Keywords:** estrogen, heart failure, menopause, mouse model, sex difference

## Abstract

Heart failure with preserved ejection fraction (HFpEF) afflicts over half of all patients with heart failure and is a debilitating and fatal syndrome affecting postmenopausal women more than any other demographic. This bias toward older females calls into question the significance of menopause in the development of HFpEF, but this question has not been probed in detail. In this study, we report the first investigation into the impact of ovary-intact menopause in the context of HFpEF. To replicate the human condition as faithfully as possible, vinylcyclohexene dioxide (VCD) was used to accelerate ovarian failure (AOF) in female mice while leaving the ovaries intact. HFpEF was established with a mouse model that involves two stressors typical in humans: a high-fat diet and hypertension induced from the nitric oxide synthase inhibitor *N*^G^-nitro-l-arginine methyl ester (l-NAME). In young female mice, AOF or HFpEF-associated stressors independently induced abnormal myocardial strain indicative of early subclinical systolic and diastolic cardiac dysfunction. HFpEF but not AOF was associated with elevations in systolic blood pressure. Increased myocyte size and reduced myocardial microvascular density were not observed in any group. Also, a broad panel of measurements that included echocardiography, invasive pressure measurements, histology, and serum hormones revealed no interaction between AOF and HFpEF. Interestingly, AOF did evoke a higher density of infiltrating cardiac immune cells in both healthy and HFpEF mice, suggestive of proinflammatory effects. In contrast to young mice, middle-aged “old” mice did not exhibit cardiac dysfunction from estrogen deprivation alone or from HFpEF-related stressors.

**NEW & NOTEWORTHY** This is the first preclinical study to examine the impact of ovary-intact menopause [accelerated ovarian failure (AOF)] on HFpEF. Echocardiography of young female mice revealed early evidence of diastolic and systolic cardiac dysfunction apparent only on strain imaging in HFpEF only, AOF only, or the combination. Surprisingly, AOF did not exacerbate the HFpEF phenotype. Results in middle-aged “old” females also showed no interaction between HFpEF and AOF and, importantly, no cardiovascular impact from HFpEF or AOF.

## INTRODUCTION

Heart failure with preserved ejection fraction (HFpEF) is an increasingly prevalent syndrome and challenge to modern cardiology. The mortality rate is comparable to that of heart failure with reduced ejection fraction (HFrEF), but unlike HFrEF therapeutic options are limited (1). The HFpEF phenotype is also skewed toward the female sex (>60% of cases), with the typical patient being a postmenopausal woman (2). Because of this demographic bias, some have called into question the possible role of menopause in the development of HFpEF (3). However, an understanding of the role of female reproductive senescence or, even more generally, the biological mechanisms underlying HFpEF has been elusive (4, 5). Progress has been hindered by challenges in recapitulating in animals the complex human phenotype (6, 7), and only recently have murine models emerged that combine the multiple comorbidities seen in patients (hypertension, obesity, diabetes, age, etc.) (8–11). Despite these advances, no model fully incorporates the influence of menopause. In addition, only associations have been made between menopause and diastolic dysfunction, a hallmark of HFpEF (12); a direct link between menopause and HFpEF has not been established. What is lacking are critical preclinical studies that can elucidate a specific role for menopause in HFpEF pathobiology (13).

In this study, we investigated the impact of reproductive senescence on HFpEF by leveraging the vinylcyclohexene dioxide (VCD) chemical model of ovary-intact accelerated ovarian failure (AOF). The VCD model approximates the human hormonal milieu during menopause transition more accurately than conventional ovariectomy, as AOF promotes a gradual decline to acyclicity while leaving ovarian androgen signaling intact. We then combined this method with a high-fat diet and nitric oxide synthase (NOS) inhibitor *N*^G^-nitro-l-arginine methyl ester (l-NAME) (8). Our new “three-hit” approach (i.e., menopause, obesity, and hypertension) enabled us to evaluate specifically how reproductive senescence altered the HFpEF phenotype in both young and middle-aged “old” female mice. To our knowledge, this report is the first preclinical study examining chemically induced AOF in the context of HFpEF.

## MATERIALS AND METHODS

### Experimental Animals and Disease Model

This study was approved by the University of Toronto Institutional Animal Care Committee (Protocol No. 20012678), and all procedures were conducted in accordance with the Canadian Council on Animal Care.

C57BL/6J mice (22 males, 61 females; Jackson Laboratories, Bar Harbor, ME) were purchased and allowed to acclimatize for at least 1 wk before inclusion in experiments. Mice denoted as “young” were 8–10 wk of age at the inception of experiments, whereas those denoted as “old” were 40–42 wk of age. The latter group corresponds to women in their midforties to early fifties, a time span when the transition to menopause naturally emerges (14). All mice were housed in groups of two to four per cage in a climate-controlled environment with a 12-h:12-h light-dark cycle. Unrestricted access to food and water, altered according to their assigned dietary regimen, was permitted for all mice throughout the study.

For each experimental arm, mice of each sex were randomly divided into two groups assigned to receive either a standard diet (chow) or the combination of a high-fat diet (HFD; D12492, Cedarlane, Burlington, ON, Canada) with l-NAME (0.5 g/L, delivered in drinking water with pH balanced to 7.5) as previously reported (8). For simplicity, we refer to the HFD and l-NAME condition as HFpEF. Female mice in each dietary regime were further divided into two groups, corresponding to vehicle control or VCD treatment (VCD) to induce AOF, resulting in four female groups (control, AOF, HFpEF, HFpEF + AOF) and two male groups (control, HFpEF). Studies in female mice were conducted in both young mice (8–10 wk of age), and older, middle-aged mice (40–42 wk of age). All male mice were 8–10 wk of age at the inception of experiments. This division resulted in the following cohort sizes of animals that progressed through the complete experimental protocol: males: 8 control, 14 HFpEF; young females: 11 control, 11 HFpEF, 11 VCD, 12 VCD + HFpEF; old females: 5 control, 2 HFpEF, 5 VCD, 4 VCD + HFpEF. Sample sizes for each statistical analysis are given in the captions of the associated figures.

During the first 18 days after acclimatization, female mice apportioned to AOF groups received daily intraperitoneal injections of VCD (94956, MilliporeSigma, Burlington, MA) delivered at 160 mg/kg via a 4 mL/kg corn oil vehicle, as previously described (15). The non-AOF female groups received injections of the corn oil vehicle control. Menopause was confirmed in all VCD-treated mice at 80 days with vaginal smears acquired daily over 10 days to confirm acyclicity.

A complementary group of male mice was randomly assigned to control or HFpEF conditions to confirm the appearance of cardiac dysfunction in male animals.

**Figure 1.**
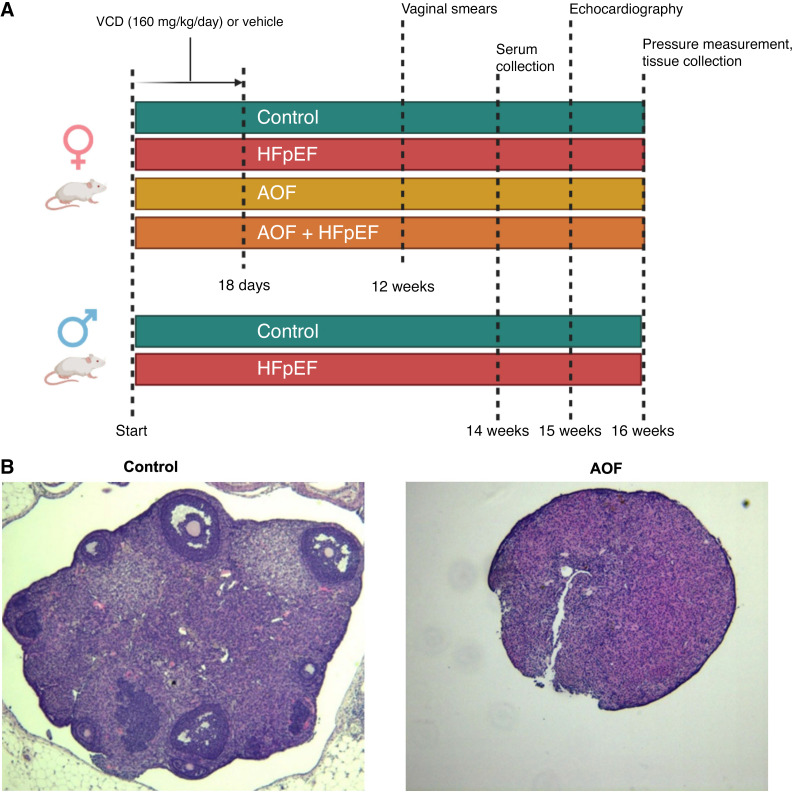
Study design and confirmation of follicle depletion following chemically induced accelerated ovarian failure (AOF). *A*: schematic of study design. *B*: representative hematoxylin-eosin (H&E)-stained midovarian sections from control and AOF mice. HFpEF, heart failure with preserved ejection fraction; VCD, vinylcyclohexene dioxide.

All groups were given their respective diets for a total of 16 wk, at which point all mice were euthanized and tissues harvested. A subset of mice was used to obtain invasive aortic and left ventricular (LV) pressure measurements through a terminal procedure. Blood sampling and detailed echocardiography were performed on all mice before study termination, at 14 and 15 wk, respectively. Figure 1*A* is a schematic of the study design.

### Echocardiography

Transthoracic echocardiography was performed with a Vevo 3100 preclinical imaging system (Fujifilm VisualSonics Inc., Toronto, ON, Canada) equipped with a 30-MHz MX400 transducer. Anesthesia was induced with 5% isoflurane (in 100% oxygen) delivered through a face mask for 3–5 min and maintained at 0.5–2.0% isoflurane. Mice were placed supine on a temperature-controlled platform. Electrocardiogram (ECG) and respiratory rate were monitored during imaging through integrated surface electrodes. Isoflurane concentration was adjusted to maintain a heart rate of 400–500 beats/min. Body temperature was monitored rectally and maintained at 36–37°C throughout data acquisition.

B-mode and M-mode recordings were acquired with a short-axis view at the midventricular level and a parasternal long-axis view visualizing the LV inflow tract. To evaluate diastolic function, pulsed-wave Doppler recordings were acquired from the LV mitral orifice at the tip level of the mitral valves in an apical four-chamber view, while tissue Doppler recordings were obtained from the septal mitral annulus. The peak velocity ratio of the early diastolic inflow wave to the mitral annulus tissue movement (E/e′) was calculated. Analysis was performed with an offline workstation with dedicated software (VevoLab; VisualSonics). All measurements were performed for a minimum of three cardiac cycles and averaged. Relative wall thickness was computed as double the posterior wall thickness measured at late diastole divided by the LV diastolic diameter (16). The myocardial performance index was determined with mitral valve pulsed-wave Doppler recordings by dividing the sum of the isovolumetric relaxation and contraction times by the aortic ejection time.

Speckle-tracking strain was analyzed from high-quality B-mode images of the parasternal long-axis and short-axis views with a minimum frame rate of 200 frames/s. For both views, the endocardium and epicardium were manually traced. Acquisitions with sections of the myocardium obscured by shadows induced by the lungs or sternal bone were excluded. The recorded respiratory tracing was used to select cardiac cycles with minimal respiratory motion for analysis (17).

### Invasive Blood Pressure Recordings

Aortic and LV blood pressure recording were acquired through a terminal experiment. Anesthesia was first induced with 5% isoflurane in 100% oxygen for 3–5 min. Mice were then positioned on a heated platform with anesthesia maintained at 3% isoflurane via a face mask. The left common carotid artery was exposed by surgical dissection and cannulated, allowing a 1.4-Fr (mouse) catheter (Millar Instruments, Houston, TX) to be inserted and advanced to the ascending aorta, where steady-state pressure recordings were acquired continuously for a minimum of 1 min. The catheter was then advanced into the LV, where pressure was again recorded for a minimum of 1 min. The data stream was recorded with the LabChart application (ADInstruments, Sydney, Australia).

### Tissue Collection

Animals were humanely euthanized via anesthesia followed by cervical dislocation. The thoracic cavity was quickly dissected to enable collection of the heart. Hearts were briefly washed with ice-cold phosphate-buffered saline (PBS) and trimmed of any pericardial tissue. Each sample was gently blotted dry and weighed before freezing on dry ice. Other tissues were then collected, weighed, and preserved in the same manner after the heart was secured.

### Histology

After tissue collection, frozen hearts were embedded in Tissue-Tek OCT compound (Sakura Finetek, Torrance, CA). Midventricular sections 7 μm thick were cut in the short axis with a cryostat (HM525 NX; Thermo Fisher, Waltham, MA) and transferred to clean slides. Immunohistochemistry was performed with primary antibodies for CD45 (70257; Cell Signaling Technologies, Danver, MA), CD68 (ab125212; Abcam, Cambridge, UK), and von Willebrand factor (vWF) (PA5-80223; Invitrogen, Waltham, MA). Fluorescent detection was completed with a goat anti-rabbit antibody conjugated with AlexaFluor 488 nm (A-11034; Invitrogen). Counterstaining with DAPI (D9542; MilliporeSigma, Burlington, MA) and AlexaFluor 647-nm-conjugated wheat germ agglutinin (WGA) (W32466; Invitrogen) was applied to all slides. Coverslips were mounted with ProLong Diamond Antifade Mountant (P36961; Invitrogen) Fluorescence imaging was performed on a FLUOVIEW FV3000 confocal-laser scanning microscope (Olympus, Tokyo, Japan) at ×20 magnification. Five to ten fields of view (FOVs) were captured from each section. Quantitative image analysis was performed with Cell Profiler (Broad Institute, Cambridge, MA). The Identify Primary Objects module with an adaptive threshold strategy and robust background method was used for counting CD45^+^, CD68^+^, and vWF puncta. For assessment of myocyte size from WGA-stained sections, FOVs showing an approximate short-axis myocyte cross section were selected. A global, minimum cross-entropy threshold was used to identify myocyte boundaries. The resulting bounded areas were measured to estimate myocyte cross-sectional area. Separately, a subset of ovaries was embedded in paraffin. Serial 5-μm sections were collected and mounted on slides to inspect the ovaries for intact follicles. To aid visualization, staining with hematoxylin and eosin (H&E) was performed as previously described (18).

### Serum Hormones

Blood was collected from the saphenous vein and allowed to fully coagulate before centrifugation (2,000 *g*, 15 min). Collection was completed in the evening hours (5:00–7:00 PM) for all mice. Fractionated serum was aspirated and stored at −80°C before downstream analysis. Samples were diluted in 5% formic acid in HPLC-grade water before liquid chromatography-mass spectrometry (LC-MS) analysis. Large molecules were then removed via spin filtration completed at 14,000 *g* for 15 min with a 3-kDa cutoff filter (MilliporeSigma, UFC5003). The resulting filtrate was vacuum dried and resuspended in 20 μL of HPLC-grade water. Relative amounts of aldosterone and corticosterone were measured with a proprietary LC-MS method that used positive-mode parallel reaction monitoring. Peak locations and linear range were identified with commercial HPLC standards and validated against the MassBank database. Relative hormone abundance was measured by comparing peak areas, which were derived by integrating a ±1-kDa window centered around each peak location.

### Statistical Analysis

Statistical analysis was performed with Python. Statistical significance was assessed by a two-way analysis of variance (ANOVA) followed by Tukey’s honestly significant difference test applied to all pairwise comparisons, as implemented in the statsmodels Python package (version 0.13.2). Box-and-whisker plots present the mean value and error bars for the 95% confidence interval. Individual biological replicates are shown as single points superimposed.

## RESULTS

### Chemical Induction of Accelerated Ovarian Failure

The impact of ovary-intact menopause via the VCD chemical model of accelerated ovarian failure (AOF) is shown in Fig. 1*B*. Whereas ovaries from control mice presented with many follicles at different stages of the menstrual cycle, follicles were entirely absent from ovaries collected from AOF mice. Daily vaginal smears collected for 10 days at 12 wk from all mice confirmed acyclicity in AOF groups.

### AOF Induces Mild Systolic and Diastolic Dysfunction Independent of HFpEF and Is Visible on Strain Imaging but Not Standard Echocardiography

**Figure 2.**
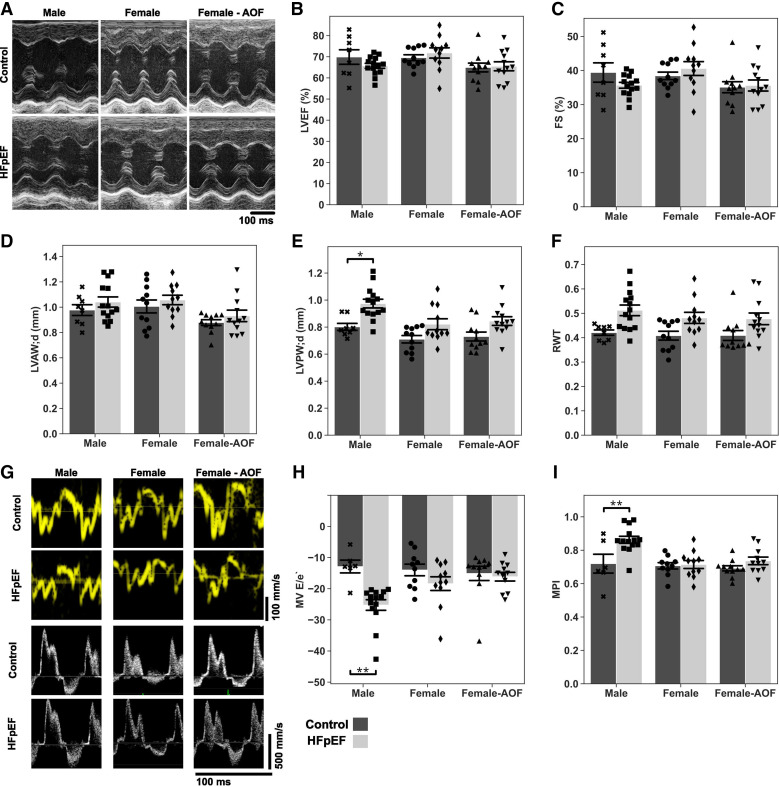
Conventional echocardiography results from mice at 15 wk. *A*: representative M-mode images acquired from a midventricular short-axis view. *B*: left ventricle ejection fraction (LVEF) percentage. *C*: fractional shortening (FS). *D*: left ventricle anterior wall thickness at late diastole (LVAW;d, in mm). *E*: left ventricle posterior wall thickness at late diastole (LVPW;d, in mm). *F*: relative wall thickness (RWT). *G*: representative tissue and pulsed-wave Doppler recordings acquired from the septal mitral annulus and mitral orifice, respectively. *H*: mitral valve (MV) ratio of the early diastolic inflow wave to the mitral annulus tissue movement (E/e′). *I*: myocardial performance index (MPI). Data are presented as means ± 95% confidence intervals with individual replicates plotted as points (*N* = 6*–*14 animals/group; *P < 0.05, **P < 0.01). AOF, accelerated ovarian failure; HFpEF, heart failure with preserved ejection fraction.

Detailed echocardiographic analysis at 15 wk to interrogate the combinatorial effects of AOF and HFpEF is shown in Fig. 2. Left ventricular ejection fraction (LVEF) and fractional shortening (FS) remained unchanged in all conditions, consistent with the HFpEF phenotype (Fig. 2, *A–C*). Male mice subjected to HFpEF alone presented with modest but significant thickening of the LV posterior wall with a concomitant increase in relative wall thickness, a marker for concentric hypertrophy (Fig. 2, *E* and *F*). LV wall thickening was absent in all female mice.

Pulsed-wave and tissue Doppler recordings were acquired from the mitral orifice and septal mitral annulus, respectively, to gauge diastolic function (Fig. 2*G*). Recordings revealed impaired diastolic function in male mice with reduced E/e′ (Fig. 2*H*) and myocardial performance index (Fig. 2*I*), whereas female mice did not display this, irrespective of AOF treatment.

**Figure 3.**
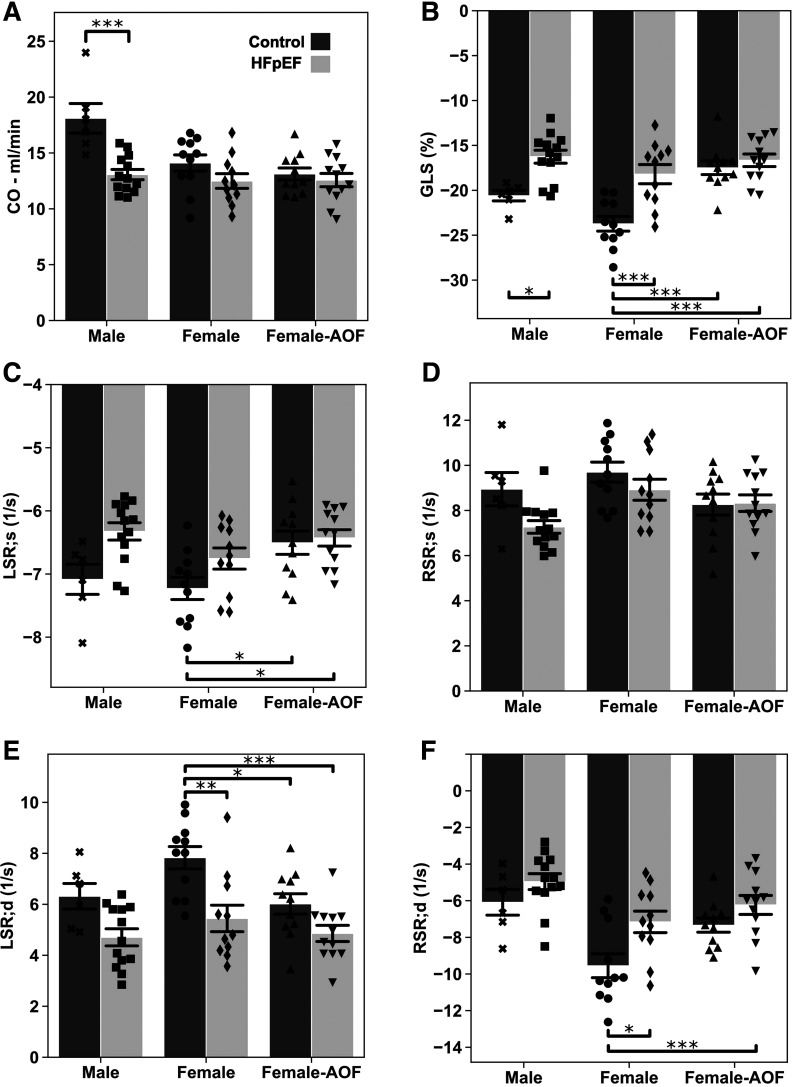
Speckle-tracking stain echocardiography results from mice at 16 wk. *A*: cardiac output (CO). *B*: global longitudinal strain (GLS) percentage. *C*: peak longitudinal systolic strain rate (LSR;s). *D*: peak radial systolic strain rate (RSR;s). *E*: peak longitudinal diastolic strain rate (LSR;d). *F*: peak radial diastolic strain rate (RSR;d). Data are presented as means ± 95% confidence intervals with individual replicates plotted as points (*N* = 6*–*14 animals/group; *P < 0.05, **P < 0.01, ***P < 0.001). AOF, accelerated ovarian failure; HFpEF, heart failure with preserved ejection fraction.

Speckle-tracking strain analysis (Fig. 3) was then performed for early detection of LV abnormalities. Strain imaging revealed the presence of reduced cardiac output (CO) in male mice subjected to HFpEF, with such changes absent in all female groups (Fig. 3*A*). In contrast, global longitudinal strain (GLS), a global measure of contractile function, was significantly reduced in all male and female HFpEF groups (Fig. 3*B*). Interestingly, AOF alone induced a comparable decline in GLS, without additional decline in the presence of HFpEF. The peak longitudinal systolic strain rate (LSR;s) saw a similar decline in mice exposed to AOF alone or AOF + HFpEF; other conditions remained unchanged (Fig. 3*C*). However, no significant change in the peak radial systolic strain rate (RSR;s, Fig. 3*D*) was observed.

Diastolic dysfunction, a principal echocardiographic finding in HFpEF, was also assessed on strain analysis. A reduced peak longitudinal diastolic strain rate (LSR;d) was observed in all female disease groups (Fig. 3*E*). As with systolic function, the addition of AOF to HFpEF did not result in a more severe phenotype than either condition alone. The peak radial diastolic strain rate (RSR;d) presented results similar to the corresponding longitudinal measure, with changes observed in all HFpEF groups but no additive effect from AOF (Fig. 3*F*).

### HFpEF, Not AOF, Elevates Aortic Systolic Blood Pressure but Does Not Impact LV Relaxation

**Figure 4.**
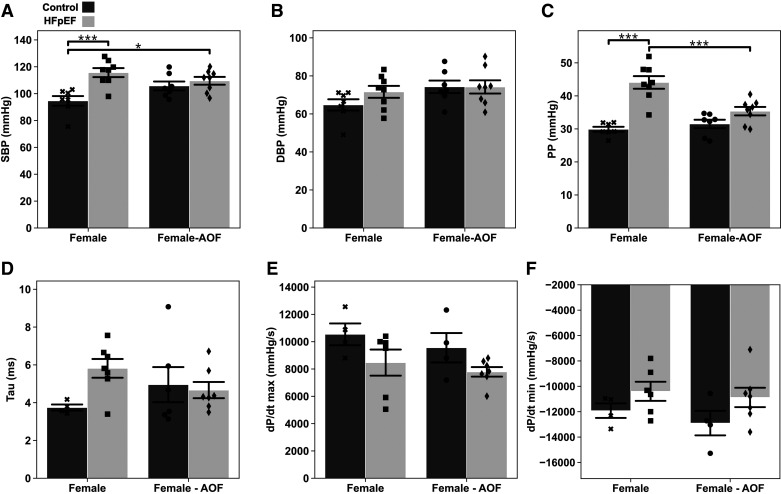
Invasive hemodynamic measurements acquired from the aorta and left ventricle. *A*: systolic blood pressure (SBP). *B*: diastolic blood pressure (DBP). *C*: pulse pressure (PP). *D*: left ventricle pressure decay constant tau (logistic model). *E*: maximum left ventricle pressure derivative with respect to time (dP/d*t*_max_). *F*: minimum left ventricle pressure derivative with respect to time (dP/d*t*_min_). Data are presented as means ± 95% confidence intervals with individual replicates plotted as points (*N* = 4*–*6 animals/group; *P < 0.05, ***P < 0.001). AOF, accelerated ovarian failure; HFpEF, heart failure with preserved ejection fraction.

Invasive assessment of hemodynamics is often considered a gold-standard tool for confirming a HFpEF diagnosis (19). We evaluated changes in blood pressure and tau (a time constant of ventricular relaxation) in a subset of female mice via pressure-volume loop measurements (Fig. 4). HFpEF mice had significantly elevated systolic blood pressure (SBP; Fig. 4*A*) but not a change in diastolic blood pressure (DBP; Fig. 4*B*). Neither measure was impacted by AOF. Whereas SBP and DBP appeared unaffected by AOF, pulse pressure (PP) was increased uniquely in mice that developed HFpEF alone, a change that was abolished by the addition of AOF (Fig. 4*C*).

LV pressure recordings were subsequently captured by advancing the catheter through the aortic valve (Fig. 4, *D*–*F*). We found equivocal evidence for changes in cardiac function in all disease groups. Curve fitting for the pressure decay constant, tau, using the logistic formulation indicated an insignificant increase in mice subjected to HFpEF (Fig. 4*D*). We recorded minimal reduction in the magnitude of the maximum and minimum pressure-time derivatives in mice subjected to HFpEF condition with or without the addition of AOF (Fig. 4, *E* and *F*).

Collectively, the observed hemodynamics in all female groups support a phenotype of cardiac dysfunction that is milder than that previously reported in male mice (8). Surprisingly, incorporating AOF into the existing two-hit model was insufficient to exacerbate the HFpEF phenotype in the female sex.

### AOF Does Not Induce Cardiac Hypertrophy or Change Vascular Density in the Context of HFpEF

**Figure 5.**
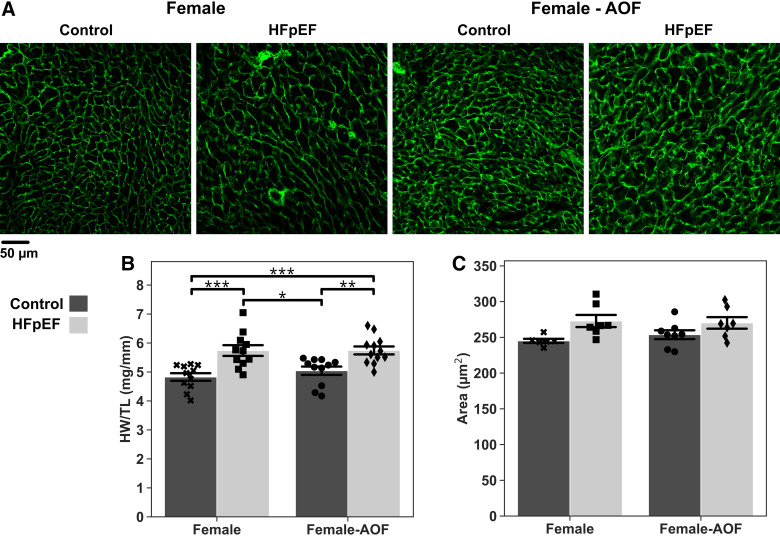
Ex vivo assessment of cardiac and myocyte hypertrophy. *A*: representative images of wheat germ agglutinin (WGA)-stained midventricular sections. *B*: heart weight-to-tibia length ratio (HW/TL). *C*: myocyte cross-sectional area. Data are presented as means ± 95% confidence intervals with individual replicates plotted as points (*N* = 6*–*12 animals/group; *P < 0.05, **P < 0.01, ***P < 0.001). AOF, accelerated ovarian failure; HFpEF, heart failure with preserved ejection fraction.

Patients with HFpEF often exhibit cardiac remodeling, including LV hypertrophy and vascular rarefaction (20, 21). Whether AOF can modify these changes is unknown. Heart weight-to-tibia length ratio (HW:TL) was measured for all female mice as a gross indicator of cardiac hypertrophy (Fig. 5). Animals subjected to HFpEF with or without AOF presented with significant and comparable increases in HW:TL, whereas such an increase was absent with AOF alone. Imaging and automated measurement of myocyte cross-sectional area uncovered similar results, with slight increases in the average myocyte size in mice exposed to HFpEF alone or in combination with AOF. Taken together, these results suggest that AOF does not alter hypertrophy.

**Figure 6.**
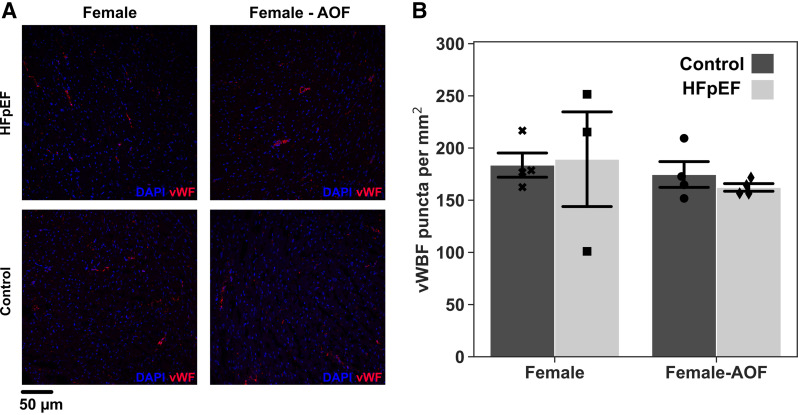
Assessment of capillary density via von Willebrand factor (vWBF) immunostaining. *A*: representative images of midventricular sections stained for vWBF with DAPI counterstaining. *B*: density of vWF puncta. Data are presented as means ± 95% confidence intervals with individual replicates plotted as points (*N* = 3 or 4 animals/group). AOF, accelerated ovarian failure; HFpEF, heart failure with preserved ejection fraction.

Vascular rarefaction was assessed on sections immunostained for von Willebrand factor (vWF), a canonical marker for platelets that allows measurement of capillary density (Fig. 6). Quantifying the density of vWF^+^ puncta, we observed no significant change in any of the groups. This represents an additional contrast from findings in male mice, where myocardial vascular rarefaction in the context of the same HFpEF model has previously been reported (8). In a fashion similar to cardiac hypertrophy (Fig. 5), microvascular remodeling in female mice was unaltered by the introduction of AOF.

### AOF, Not HFpEF, Increases Myocardial Leukocyte Concentration

**Figure 7.**
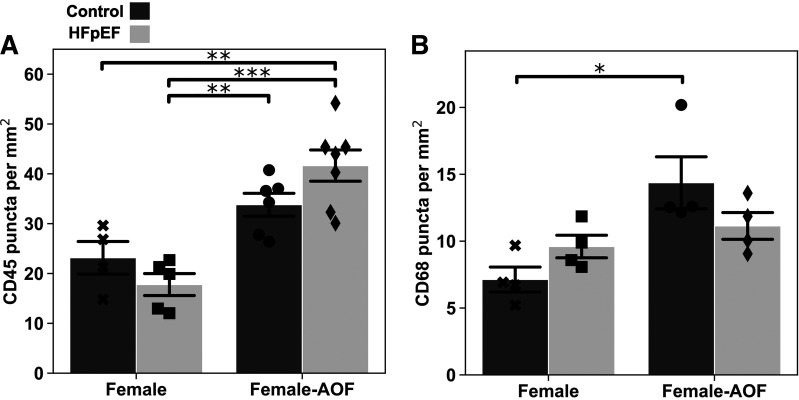
Evaluation of infiltrating leukocytes in the myocardium via immunostaining of midventricular section. *A*: density of CD45^+^ puncta. *B*: density of CD68^+^ puncta. Data are presented as means ± 95% confidence interval with individual replicates plotted as points (*N* = 5 animals/group; *P < 0.05, **P < 0.01, ***P < 0.001). AOF, accelerated ovarian failure; HFpEF, heart failure with preserved ejection fraction.

Endomyocardial biopsies have previously uncovered evidence for an increased presence of infiltrating immune cells in human HFpEF hearts (20). We examined whether this feature is recapitulated by our model and whether it may be implicated in the effects of AOF. Immunostained cryosections obtained from female mouse hearts are shown in Fig. 7. Staining for the pan-leukocyte surface marker CD45 revealed an increased presence of positive puncta because of AOF alone or in combination with HFpEF. Such an increase was absent in mice subjected to HFpEF alone. Staining for the macrophage marker CD68 demonstrated similar patterns, with the application of AOF with or without HFpEF resulting in an increased abundance of CD68^+^ puncta. These results suggest that AOF may promote an inflammatory response and increase leukocyte infiltration into myocardium independent of HFpEF. This elevated presence of immune cells provides a potential explanation for the subclinical cardiac dysfunction observed in female mice subjected to AOF alone.

### Circulating Corticosteroids Are Elevated by HFpEF and Unaltered by AOF

Increased mineralocorticoid signaling is an established feature and therapeutic target in heart failure, with a known link to menopause (22–24). Elevated glucocorticoid signaling has previously been posited as a causal factor in the emergence of metabolic dysfunction in ovariectomized mice (25).

**Figure 8.**
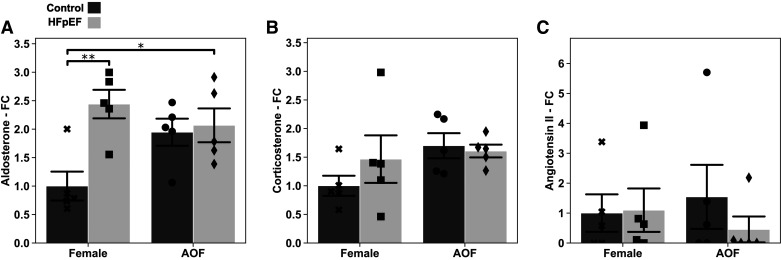
Relative levels of select circulating hormones measured on tandem liquid chromatography-mass spectrometry. Fold changes (FCs) for each hormone are given relative to the mean value for the ovary-intact control group. *A*: aldosterone fold change. *B*: corticosterone fold change. *C*: angiotensin II fold change. Data are presented as means ± 95% confidence intervals with individual replicates plotted as points (*N* = 5 animals/group; *P < 0.05, **P < 0.01). AOF, accelerated ovarian failure; HFpEF, heart failure with preserved ejection fraction.

To explore a possible contribution from these axes, we measured the abundance of both serum aldosterone and corticosterone using tandem LC-MS (Fig. 8). As anticipated, serum aldosterone was significantly elevated in both HFpEF and HFpEF + AOF groups: AOF alone led to an increase of borderline significance (Fig. 8*A*). A nonsignificant increase in corticosterone was observed in all disease groups, again with no evidence for additive effects for AOF alongside HFpEF (Fig. 8*B*). Levels of angiotensin II, a canonical player in the neurohormonal activation often observed in heart failure, were below the level of detection in many samples and presented no perceptible changes between groups (Fig. 8*C*).

### Age Does Not Exacerbate the Cardiovascular Effects of AOF

**Figure 9.**
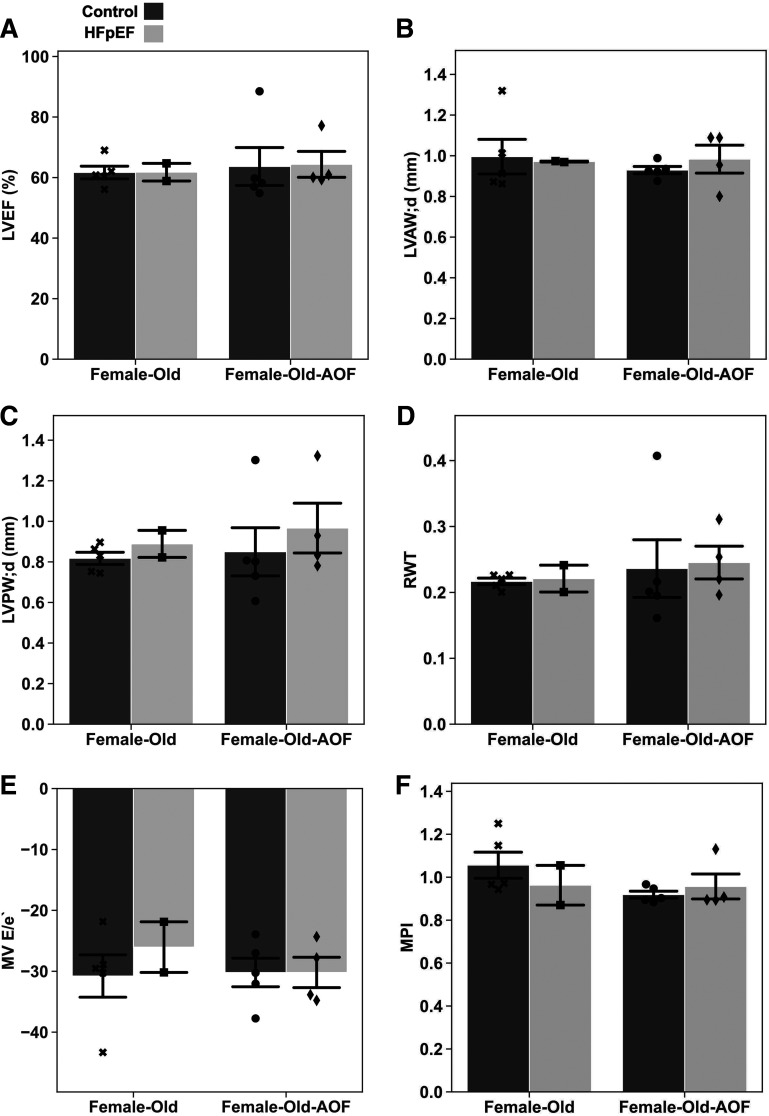
Conventional echocardiography results from middle-aged “old” female mice at 15 wk from study inception. *A*: left ventricle ejection fraction (LVEF) percentage. *B*: left ventricle anterior wall thickness at late diastole (LVAW;d, in mm). *C*: left ventricle posterior wall thickness at late diastole (LVPW;d, in mm). *D*: relative wall thickness (RWT). *E*: mitral valve (MV) ratio of the early diastolic inflow wave to the mitral annulus tissue movement (E/e′). *F*: myocardial performance index (MPI). Data are presented as means ± 95% confidence intervals with individual replicates plotted as points (*N* = 2*–*5 animals/group). AOF, accelerated ovarian failure; HFpEF, heart failure with preserved ejection fraction.

It is conceivable that an interaction between reproductive senescence and HFpEF is obscured in the preceding results because of the relatively young age of the animals. To evaluate this possibility and determine whether the impact of AOF in the context of HFpEF may be considerably modified by animal age, we replicated the experiments performed in female mice using middle-aged “old” female mice. The induction of AOF, application of HFpEF, and all other details pertaining to animal handling were reprised, with the sole exception of the use of older mice, corresponding to women in the midforties to early fifties, at the initiation of experiments. Again, cardiac function was first assessed by conventional echocardiography (Fig. 9). Consistent with our findings in younger female mice, neither HFpEF, AOF, nor the combination produced any change in LVEF (Fig. 9*A*). Age similarly did not modulate changes in the thickness of either the anterior or posterior LV wall or the relative wall thickness (Fig. 9, *B–D*). Finally, changes in diastolic function arising from any experimental condition were also absent on conventional echocardiography (Fig. 9, *E* and *F*). These results discredit the possibility that age has a significant capacity to exacerbate the cardiovascular impact of AOF or HFpEF in this model.

**Figure 10.**
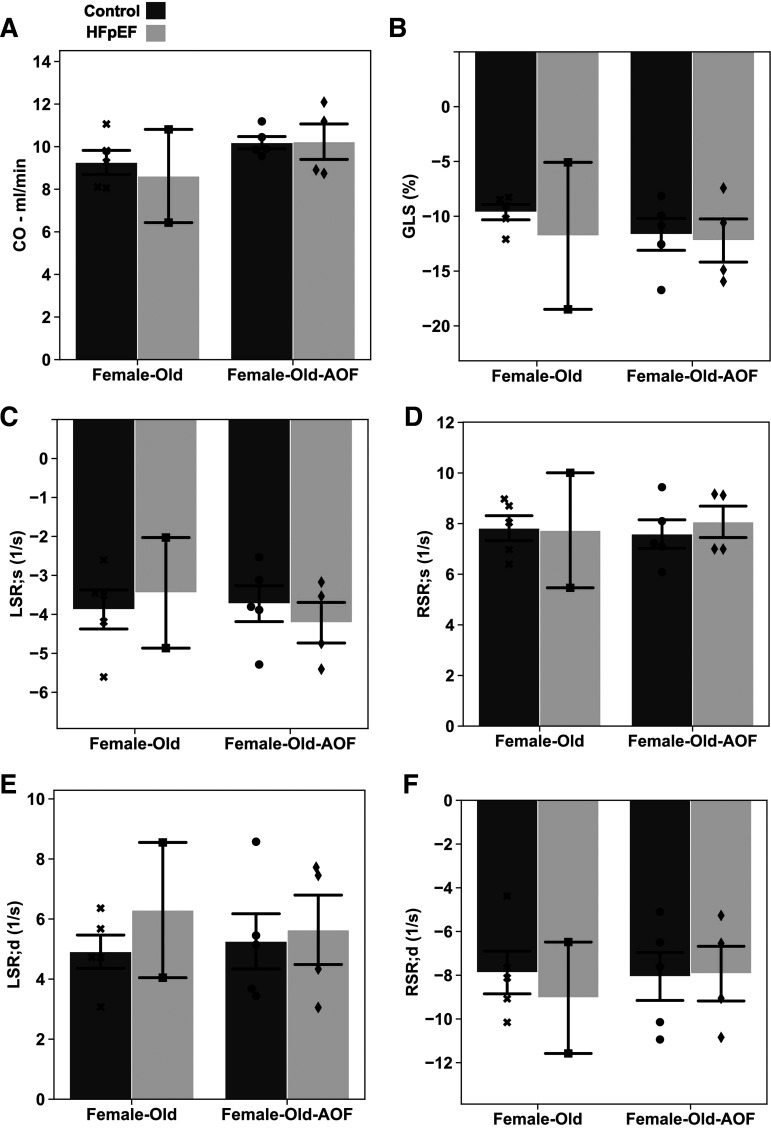
Speckle-tracking stain echocardiography results from middle-aged “old” female mice at 15 wk from study inception. *A*: cardiac output (CO). *B*: global longitudinal strain (GLS) percentage. *C*: peak longitudinal systolic strain rate (LSR;s). *D*: peak radial systolic strain rate (RSR;s). *E*: peak longitudinal diastolic strain rate (LSR;d). *F*: peak radial diastolic strain rate (RSR;d). Data are presented as means ± 95% confidence intervals with individual replicates plotted as points (*N* = 2*–*5 animals/group). AOF, accelerated ovarian failure; HFpEF, heart failure with preserved ejection fraction.

A more sensitive examination was achieved with speckle-tracking strain analysis of the LV (Fig. 10), in the same manner applied to young mice (Fig. 3). Results of this analysis were summarily contradictory to findings obtained in young mice: in contrast to the subtle cardiac dysfunction present in young HFpEF, AOF, and HFpEF + AOF females, older female mice displayed equivocal evidence for any abnormality. Measures of cardiac output (Fig. 10*A*), global strain (Fig. 10*B*), and peak global strain rates during both systole and diastole (Fig. 10, *C–F*) were unaltered.

**Figure 11.**
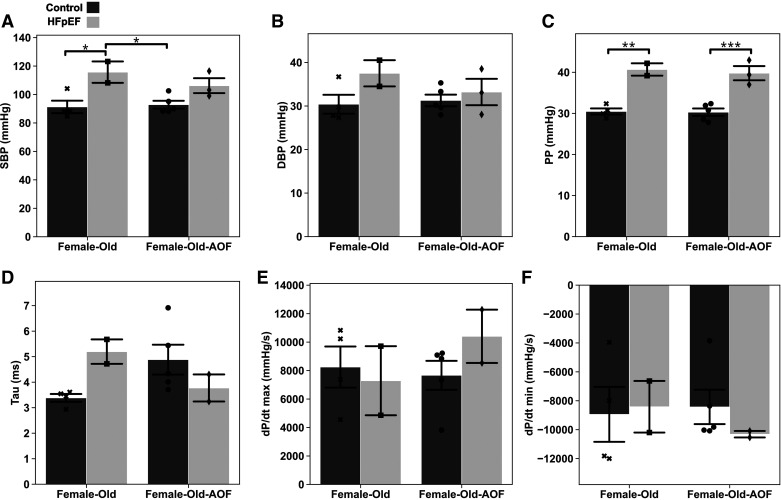
Invasive hemodynamic measurements acquired from the aorta and left ventricle of middle-aged “old” female mice. *A*: systolic blood pressure (SBP). *B*: diastolic blood pressure (DBP). *C*: pulse pressure (PP). *D*: left ventricle pressure decay constant tau (logistic model). *E*: maximum left ventricle pressure derivative with respect to time (dP/d*t*_max_). *F*: minimum left ventricle pressure derivative with respect to time (dP/d*t*_min_). Data are presented as means ± 95% confidence intervals with individual replicates plotted as points (*N* = 2*–*5 animals/group; *P < 0.05, ***P < 0.001). AOF, accelerated ovarian failure; HFpEF, heart failure with preserved ejection fraction.

Finally, invasive pressure-volume loop measurements were obtained from older females via invasive catheterization (Fig. 11). Here, both AOF and control females subjected to HFpEF exhibited increases in SBP (Fig. 11*A*) and DBP (Fig. 11*B*), although only the increase in SBP in HFpEF-only mice rose to statistical significance. PP was significantly elevated in all females subjected to HFpEF but was not meaningfully altered by AOF (Fig. 11*C*). Changes in LV hemodynamics were equivocal, with no clear changes in terms of the tau constant (Fig. 11*D*) or the maximum or minimum pressure-time derivatives (Fig. 11, *E* and *F*). In sum, invasive pressure recordings in older females revealed modest hypertension unmodulated by AOF.

## DISCUSSION

Clinical management of HFpEF is a growing challenge to modern health care. Contributing to this are the vast heterogeneity of the condition, a poor understanding of the underlying pathobiology, and limited therapeutic options. In humans, it is well recognized that most patients with HFpEF are women, and large epidemiological studies have established associations between early menopause and both higher risk of cardiovascular disease (26) and divergent LV remodeling (27). This preponderance of women with HFpEF is beginning to be reflected in the preclinical literature, with multiple studies explicitly incorporating female sex as a feature of the animal model used (11, 28, 29). However, in the current preclinical landscape, the impact of reproductive senescence in HFpEF has not been well modeled, which impedes further uncovering of whether and how menopause shapes the HFpEF syndrome.

Elucidating the dynamics of menopause and its influence on cardiovascular health is challenging given its gradual and complex nature. A reliable animal model is necessary to acquire actionable, mechanistic insights. Rodents transition to acyclicity in a starkly different fashion, notably including a period of continuous excess estrogen production rather than the monotonic decline seen in humans. Ovariectomy, the traditional experimental approach to replicating human menopause, is flawed in that it avoids both the kinetics and hormonal milieu of physiological menopause (30, 31) and abolishes endocrine signaling that persists long past menopause, particularly the secretion of androgens that have been associated with increased cardiovascular risk (32–35). Similarly, preclinical modeling of HFpEF itself is subject to difficulties; the phenotype typically emerges in the context of multiple systemic comorbidities over a prolonged time frame. Many early purported models of HFpEF fail to integrate this multifaceted nature and bear only limited fidelity with the human condition (6, 7). In the case of both HFpEF and menopause, as well as their combination, faithful modeling requires a holistic approach.

In this study, we endeavored to accurately represent a feature common in the medical history of the typical patient with HFpEF: a gradual transition to ovary-intact menopause. Our approach leverages the VCD model of chemically induced AOF, a potent and well-established tool arising from years of research in reproductive and endocrine biology (31, 36, 37). The VCD model engenders a gradual transition to reproductive senescence. To understand how AOF may interact with HFpEF, we employed a state-of-the-art, two-hit mouse model combining a high-fat diet with l-NAME (8). To our knowledge, this work represents the first study to investigate how ovary-intact AOF impacts disease presentation in a bona fide preclinical model of HFpEF. Our study leverages gold-standard preclinical tools for evaluating cardiovascular health, including high-frequency echocardiography with speckle-tracking strain imaging and invasive hemodynamic measurements.

Before the key findings of this study are summarized, it is instructive to note that previous works examining sex differences in our selected mouse model of HFpEF have been inconsistent. Reports asserting that female sex affords protection from the HFpEF phenotype (28) contrast starkly against those demonstrating significant HFpEF symptoms in female mice (29). We anticipated that the introduction of AOF would worsen the cardiac dysfunction and remodeling achieved with the standard two-hit HFpEF model. This assumption was founded on a large body of literature detailing the cardioprotective effects of estrogen signaling in both premenopausal humans and mice (38, 39). Contrary to this hypothesis, our findings revealed an incremental impact in young female mice from adding AOF to the two-hit HFpEF model. When compared with frank cardiac dysfunction in male mice, diastolic and systolic dysfunction in young female mice with HFpEF, with or without AOF, or with AOF alone was subclinical and detected only on strain imaging, thus suggesting that female sex can confer some protection independent of reproductive status.

The absence of a sensitizing effect from AOF in a two-hit model of HFpEF stands in contrast to effects observed in other experimental cardiovascular insults. VCD-treated mice exhibit more pronounced hypertension in response to angiotensin II infusion (40). AOF also engenders greater infarct sizes relative to controls in response to cardiac ischemia-reperfusion (15). One explanation for the discrepancy between these findings and our own results may be the relatively acute nature of these models compared with the protracted character of the HFpEF model we used. Another possible explanation is the different time points examined in the work of Pollow et al. (40) and Konhilas et al. (15). These previous studies considered the perimenopausal period, whereas the present study considers strictly the early postmenopausal period. The distinct hormonal milieus in the perimenopausal versus postmenopausal period may very likely present different stressors to the heart.

Concentric cardiac hypertrophy is a frequently encountered form of cardiac remodeling in human HFpEF. Although there was some evidence for both increased LV wall thickness and modest myocyte hypertrophy in the HFpEF heart, we observed that AOF did not modify this in any of these measures in young female mice. This concurs with preceding works reporting an unaltered hypertrophic response in VCD-treated mice subject to either ANG II infusion (15) or voluntary exercise (41).

Although AOF did not overtly sensitize mice to HFpEF, mild, subclinical cardiac dysfunction due to the loss of ovarian function alone was clearly observed. The impact of AOF thus extends to the heart and cannot definitively be dismissed as orthogonal to HFpEF. This assertion is supported by our finding that AOF bolsters the presence of infiltrating leukocytes in the myocardium. Multiple lines of evidence have also implicated altered immune cell dynamics in HFpEF. Staining of HFpEF endomyocardial biopsies has revealed an elevated presence of CD68^+^ macrophages (20). Blood collected from patients with HFpEF has revealed an increased number of neutrophils, a shift mimicked by an increase in cardiac neutrophils in the hearts of HFpEF mice (42). In parallel, a sizable body of work has linked menopause to changes in the human immune system (43, 44). AOF may represent a useful tool to capture such interactions that are potentially salient in HFpEF. Indeed, induced differences in ANG II-induced hypertension have previously been attributed to shifts in T-cell populations and behavior (40, 45). Ultimately, the interplay of menopause and inflammation and their impact on cardiac function remain poorly characterized. Like previous literature findings, our results demonstrate an association among menopause, inflammation, and cardiac dysfunction. It is unlikely that estrogen deprivation alone directly impacts the heart. One possible mechanism is that without estrogen downregulation of inflammatory cytokines, the postmenopause state promotes systemic inflammation, which increases the risk of cardiac dysfunction. However, exactly how inflammation contributes to cardiac dysfunction remains unknown. Furthermore, why infiltrating leukocytes were absent in the myocardium of non-AOF HFpEF mice is also unclear.

Aberrant renin-angiotensin-aldosterone system (RAAS) signaling is a hallmark of heart failure. In HFpEF aldosterone, secreted via the adrenal glands, is frequently elevated. Mineralocorticoid receptor antagonists targeting this signaling have been deployed as a therapy for HFpEF and have shown some success in several clinical trials. In ovariectomized mice, subsequent metabolic remodeling is accompanied by high levels of circulating corticosteroids and increased glucocorticoid sensitivity and can be abolished by concurrent removal of the adrenal glands (25). We explored the possibility of cross talk between AOF and increased adrenal signaling through direct measurement of serum aldosterone and corticosterone by LC-MS. Aldosterone concentrations were elevated in the context of HFpEF but remained unchanged with the addition of AOF. An increase due to AOF alone was also recorded, although not rising to significance. Remodeling of corticosteroid signaling because of menopause remains possible and warrants further investigation as a salient feature of HFpEF.

In considering the importance of menopause in the etiology of HFpEF, one questions whether effects of any importance can be wholly decoupled from age. Observing that AOF alone did not appreciably worsen the HFpEF phenotype, despite provoking changes in myocardial immune cell populations, we hypothesized that an advanced age may be required to make the full physiological consequences of AOF apparent. To test this hypothesis, we recapitulated aspects of the original study using middle-aged “old” female mice. When compared with young female control mice, old female control mice clearly exhibited features of an aged albeit still healthy heart [e.g., decreased LVEF, relative wall thickness (RWT), CO, GLS]. Interestingly, neither echocardiography nor invasive pressure recordings indicated evidence for an interaction between HFpEF and AOF, suggesting that the effects of AOF were independent of age. Furthermore, the older female heart was remarkably robust against the negative cardiovascular ramifications seen in young females from either HFpEF or AOF. This observation begs revisiting the hypothesis that AOF is a primary culprit underlying the bias toward postmenopausal women in the HFpEF population; other as yet undiscovered influences are almost certainly at play. It is entirely possible that had an even older cohort of mice (18–24 mo of age) been included the signs and symptoms of HFpEF would be pronounced, and these would arise from either old age or the chronic depletion of estrogen and its downstream effects or both. What we can conclude from the results of this study is that AOF in young mice (early menopause) brought on cardiac changes similar to those from HFpEF stressors alone but that AOF in older mice (middle-aged women going through normal menopause) did not. The findings beg further investigation of the interplay between menopause and age of onset.

This inconsistency together with the absent to mild HFpEF phenotype in female mice across both age and reproductive status foments broader concerns regarding the strength of the HFD + l-NAME combination to induce HFpEF. Although several works have leveraged this approach, phenotype severity does vary markedly (8, 46–48). One work in particular tested 30 inbred strains of mice using this protocol and reported large strain-to-strain variability along with inconsistent sex differences (29). In this context, we submit that the HFD + l-NAME model of HFpEF can induce a severe HFpEF phenotype, but this ability appears inconsistent and not wholly reliable.

There exist some specific limitations to our study that warrant noting for further contextualization of our findings. The use of l-NAME, although widely adopted for inducing experimental hypertension, does not impart the volume overload common in symptomatic patients with HFpEF. Chronic treatment with l-NAME may also induce a compensatory upregulation in the proinflammatory isoform induced NOS (iNOS), biasing the inflammatory milieu (49). Another limitation is the relatively short duration of this study: although the 16-wk interval is comparable to other work using the two-hit model, it is nonetheless a dramatically compressed timeline for disease development and is a sharp contrast to the multidecade process underlying human HFpEF. From this compressed timeline, it is reasonable to expect only the emergence of the earliest cardiac changes, as seen on strain imaging, but not later ones, such as significant hypertrophy. A third limitation is that we did not identify the exact time of ovarian failure, only that at 80 days ovarian failure had already occurred. Future investigations will determine whether the duration of a postfailure state bears relevance to the findings reported here. A related consideration is that if the postfailure state is, indeed, recent, then the 4-wk post-ovarian failure interval examined in the present study will not inform on the long-term impact relevant in women 60 yr of age and older. In fact, a minimum of 12 wk after ovarian failure in VCD mice is necessary to probe the chronic cardiac ramifications of estrogen deficiency. Finally, we cannot rule out with absolute certainty potential off-target effects from VCD treatment. As assiduous investigation of VCD’s impacts has yielded minimal evidence for effects beyond the ovaries, we submit that attributing our findings to the accelerated loss of ovarian follicles is most plausible (50–53).

Considering the prevalence of physiological menopause in the human HFpEF population together with the purported necessity for improved precision in clinical management, a better understanding of the role of menopause in HFpEF pathobiology is desirable. As we have demonstrated, incorporating reproductive senescence without recourse to a surgical procedure is feasible and introduces effects pertinent to heart failure. Future research trained on forming a comprehensive understanding of HFpEF etiology and cardiovascular disease more generally should consider integrating AOF to represent the more realistic elderly female patient.

We have presented, to our knowledge, the first targeted study exploring the effects of menopause through accelerated ovarian failure in the context of HFpEF. Contrary to expectation, AOF did not meaningfully sensitize mice to a model of HFpEF in early-stage menopause. AOF independently induces measurable and significant changes in cardiac function in the absence of cardiac hypertrophy or changes in blood pressure. AOF stimulates infiltration of leukocytes into the myocardium, an established feature of human HFpEF with emerging significance. Interestingly, an interaction between AOF and the effects of HFpEF does not appear to be modulated by age; cardiac dysfunction due to AOF alone was observed only in young mice but not middle-aged old mice. We submit that our findings represent essential early steps in explaining sex differences in HFpEF and constructing a more nuanced understanding of this increasingly common syndrome.

## DATA AVAILABILITY

Data are available upon request to H.-L. M. Cheng (hailing.cheng@utoronto.ca).

## GRANTS

This work was supported by Canadian Institutes of Health Research (CIHR) Canada Graduate Scholarship Doctoral Award CGS-D (to A.M.T.), CIHR Grant PJT 175131 (to H.-L.M.C.), Natural Sciences and Engineering Research Council of Canada Grant 2019–06137 (to H.-L.M.C.), and Canada Foundation for Innovation Ontario Research Fund Grant 34038 (to H.-L.M.C.).

## DISCLOSURES

No conflicts of interest, financial or otherwise, are declared by the authors.

## AUTHOR CONTRIBUTIONS

A.M.T. conceived and designed research; A.M.T., D.N., D.G., A.M., Y.-Q.Z., and M.M. performed experiments; A.M.T., A.M., and Y.-Q.Z. analyzed data; A.M.T., F.B., and H.-L.M.C. interpreted results of experiments; A.M.T. prepared figures; A.M.T. drafted manuscript; A.M.T., Y.-Q.Z., M.H., F.B., and H.-L.M.C. edited and revised manuscript; A.M.T., D.N., D.G., A.M., Y.-Q.Z., M.M., M.H., F.B., and H.-L.M.C. approved final version of manuscript.
